# To Transfuse or Not to Transfuse: A Case of Unresectable Renal Cell Carcinoma-Induced Warm Autoimmune Hemolytic Anemia

**DOI:** 10.7759/cureus.48345

**Published:** 2023-11-06

**Authors:** Ellen Yos, Austin Patrick-Eisenberg, Jamie Campbell

**Affiliations:** 1 Internal Medicine, Grand Strand Medical Center, Myrtle Beach, USA

**Keywords:** hemodynamic instability, unresectable malignancy, renal cell carcinoma, transfusion threshold, warm autoimmune hemolytic anemia

## Abstract

Warm autoimmune hemolytic anemia (WAIHA) is a rare disease. Roughly half of all cases are considered either primary or idiopathic. The remaining cases are typically secondary to a drug reaction or an underlying disease state such as malignancy, infection, or chronic autoimmune disease. Treatments for WAIHA include corticosteroids, intravenous immunoglobulin (IVIG), rituximab, and splenectomy. We present a case of WAIHA with underlying clear cell renal cell carcinoma (RCC) that was unresectable, creating a difficult treatment course.

A 76-year-old male with recently diagnosed clear cell RCC was admitted with symptomatic WAIHA and significant hemodynamic instability. Over the course of his admission, he received 25 blood transfusions, erythropoietin, methylprednisolone, IVIG, rituximab, and mycophenolate mofetil in an attempt to control his disease state. He suffered end-organ damage in the form of heart failure with reduced ejection fraction. He was deemed too unstable for RCC resection or interventional cardiac procedures. Determining an appropriate transfusion threshold proved to be a noteworthy challenge. His hemoglobin eventually stabilized to 7.4 g/dL upon discharge over the course of 27 days of treatment. The underlying cause of his WAIHA was believed to be most likely secondary to RCC.

WAIHA may have a prolonged treatment course with high risk of mortality if the underlying cause is not resolvable. If this is the case, it can be difficult to determine a hemoglobin transfusion threshold that maintains normal vital signs while minimizing the risk of transfusion-associated circulatory overload (TACO) and transfusion-related acute lung injury (TRALI). Prolonged hemodynamic instability may result in end-organ damage. For our patient, we aimed for a hemoglobin transfusion threshold of 5.0-6.0 g/dL based on his mean arterial pressure (MAP), heart rate, and subjective symptoms.

## Introduction

While warm autoimmune hemolytic anemia (WAIHA) is a rare disease with an incidence of 10 to 30 in one million, it is still the most common form of autoimmune hemolytic anemia (AIHA), comprising approximately 70% of all cases [1-3]. There is a wide range of WAIHA etiologies, with half of all cases separated into primary or idiopathic and the other half described as being secondary to an underlying disorder [2-4]. Broad categories of these underlying disorders include infection, malignancy, lymphoproliferative disorders, autoimmune diseases, and drug-induced [5]. In general, immunosuppressive medications such as systemic glucocorticoids, monoclonal antibodies, intravenous immunoglobulin (IVIG), and antimetabolites are used for supportive treatment, while splenectomy can be the last resort in patients who are non-respondent to immunosuppressants. Whenever possible, the underlying cause of WAIHA should be determined and addressed. Circulatory support should be provided in the form of blood transfusions and intravenous (IV) fluids. A prior retrospective study of hospitalized AIHA patients performed by Chen et al. determined that the transfusion threshold that minimized transfusion reactions while maintaining systemic perfusion was a hemoglobin of 4.0-5.0 g/dL [6]. Other expert opinion recommends transfusion for hemoglobin less than 6.0 g/dL or when the patient is symptomatic [7]. Here we present a case of WAIHA in a patient with a history of renal cell carcinoma (RCC), unresectable due to severe hemodynamic instability, and discuss the issue of red blood cell transfusion threshold.

## Case presentation

The patient was a 76-year-old male with a history of clear cell RCC diagnosed in 2022 for which he was not currently receiving treatment. He also reported a one-month history of tension headaches, which he had been treating with over-the-counter ibuprofen 800 mg once daily. His baseline hemoglobin prior to admission was 14.0 - 15.0 g/dL. The patient presented to the hospital six months after RCC diagnosis with progressively worsening shortness of breath, generalized weakness, tachycardia, and tachypnea for three days. Initial vital signs on the evening of hospital day one, obtained by the admitting night team, showed an oral temperature of 99.8^o^F (37.6^o^C), sinus tachycardia with a rate of 118 beats per minute, respiratory rate of 22 breaths per minute, blood pressure 113/64 mmHg, oxygen saturation of 98% on room air. 

The physical exam performed by the day team on the morning of hospital day two revealed a patient who was acutely ill-appearing, diaphoretic, tachypneic, in moderate distress secondary to dyspnea without use of accessory muscles. Heart sounds were tachycardic with a regular rhythm and a systolic ejection murmur heard at all points of cardiac auscultation, but best heard at the left upper sternal border. There was jugular venous distention to just below the mandible with patient lying at a 45-degree angle. Mucous membranes were dry. Lungs were clear to auscultation bilaterally without wheezes, rales, or rhonchi noted. The abdomen was soft, non-tender, and non-distended, with normoactive bowel sounds in all four quadrants. Extremities appeared grossly unremarkable without pitting edema or peripheral cyanosis. Neurological exam showed no focal weakness with cranial nerves grossly intact. The skin was noted to be jaundiced with pale conjunctival membranes.

On admission, basic labs including a complete blood count (CBC), complete metabolic panel (CMP), arterial blood gas (ABG), lactic acid, lactate dehydrogenase (LDH), reticulocyte count, haptoglobin, prothrombin time (PT), and international normalized ratio (INR) were obtained (tabulated results can be found in Tables 1-5).

**Table 1:** Results of complete blood count on hospital day one. CBC (complete blood count), WBC (white blood cells), RBC (red blood cells), HGB (hemoglobin), HCT (hematocrit), MCV (mean cell volume), PLT (platelets).

CBC		Reference range
WBC	9.4	3.7 - 10.1 K/mm3
RBC	1.92	4.55 - 5.47 M/mm3
HGB	5.8	14.0 - 16.4 g/dL
HCT	16.6	40.0 - 47.2 %
MCV	86.6	81.8 - 94.6 fL
PLT	173	150 - 400 K/mm3

**Table 2:** Results of complete metabolic panel on hospital day one. CMP (complete metabolic panel), BUN (blood urea nitrogen), AST (aspartate aminotransferase), ALT (alanine aminotransferase).

CMP		Reference range
Sodium	145	135 - 146 mmol/L
Potassium	3.7	3.5 - 5.1 mmol
Chloride	106	96 - 107 mmol/L
Carbon dioxide	28	22 - 32 mmol/L
Anion gap	10	3.0 - 11.0 mEq/L
BUN	41	7.0 - 20.0 mg/dL
Creatinine	0.8	0.7 - 1.5 mg/dL
Glucose	148	74 - 106 mg/dL
Calcium	9.5	8.4-10.2 mg/dL
Total bilirubin	13.1	0.1 - 1.1 umol/L
Direct bilirubin	1.1	0.0 - 0.2 umol/L
AST	66	15 - 46 Units/L
ALT	40	13 - 69 Units
Alkaline phosphatase	137	38 - 126 Units/L

**Table 3:** Results of miscellaneous lab testing on hospital day one.

Miscellaneous		Reference range
Lactate dehydrogenase	446	100 -190 units/L
Lactic acid	3.9	0.7 - 2.1 mmol/L
Reticulocyte Count	0.0848	0.02 - 0.11 mill/mm3
Reticulocyte %	4.3	0.5 - 1.5 %
Haptoglobin	< 10	34 - 355 mg/dL

**Table 4:** Results of arterial blood gas on hospital day one. ABG (arterial blood gas), pCO2 (partial pressure of carbon dioxide), pO2 (partial pressure of oxygen), HCO3 (bicarbonate).

ABG		Reference range
pH	7.454	7.35 - 7.45
pCO2	26.3	35.0 - 48.0 mmHg
pO2	74.7	83.0 - 108.0 mmHg
HCO3	18.4	21 - 28 mmol/L

**Table 5:** Results of coagulation panel on hospital day one. PT (prothrombin time), INR (international normalized ratio).

Coagulation panel		Reference range
PT	12.3	9.8 - 13.9 seconds
INR	1.07	0.9 - 1.1

He was found to have a hemoglobin of 5.4 g/dL, total bilirubin of 13.1 umol/L, direct bilirubin of 1.1 umol/L, initial lactate dehydrogenase of 446 units/L, haptoglobin less than 10 mg/dL, absolute reticulocyte count of 0.0848 mill/mm3, reticulocyte percentage 4.3%, and reticulocyte production index (RPI) 0.6%.

ABG obtained while the patient was on room air showed pH 7.454, partial pressure of carbon dioxide (pCO2) 26.3 mmHg, partial pressure of oxygen (pO2) 74.7 mmHg, and bicarbonate (HCO3) 18.4 mmol/L. Other findings on the CBC included white blood cell (WBC) count 9.4 K/mm3, red blood cell (RBC) count 1.92 M/mm3, hematocrit (HCT) 16.6%, mean cell volume (MCV) 86.6 fL, and platelets 173 K/mm3.

The remainder of the complete metabolic panel revealed sodium 145 mmol/L, potassium 3.7 mmol, chloride 106 mmol/L, carbon dioxide 28 mmol/L, anion gap 10 mEq/L, blood urea nitrogen (BUN) 41 mg/dL, creatinine 0.8 mg/dL, glucose 148 mg/dL, calcium 9.5 mg/dL, aspartate aminotransferase (AST) 66 Units/L, alanine aminotransferase (ALT) 40 Units, and alkaline phosphatase 137 Units/L. Lactic acid was 3.9 mmol/L. PT was 12.3 seconds and the INR was 1.07. Urinalysis revealed 4 mg/dL of urobilinogen (0.0-1 mg/dL) but was otherwise within normal limits.

**Figure 1:**
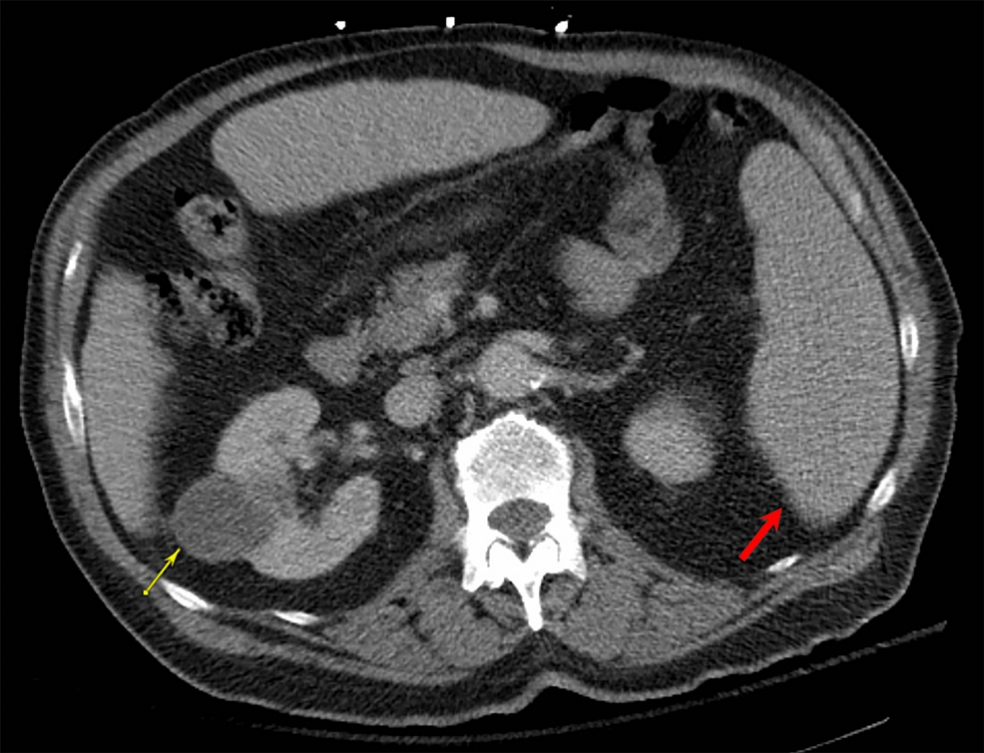
CT abomen and pelvis with IV contrast demonstrating splenomegaly (red arrow) and a right interpolar renal lesion (yellow arrow) measuring 2.7 cm consistent with renal cell carcinoma.

At this early juncture in the hospital course, there was a high suspicion for a hemolytic anemia given his low hemoglobin, red blood cell count, hematocrit, and haptoglobin, combined with his elevated LDH and elevated indirect bilirubin of 12.0 umol/L (total bilirubin 13.1 umol/L minus direct bilirubin 1.1 umol/L). To investigate this further, he underwent a series of lab and imaging tests to determine the etiology of the suspected underlying hemolytic anemia, including causes of AIHA. His direct antiglobulin test (DAT) was positive for warm autoantibodies, and his CT abdomen and pelvis showed splenomegaly and a right interpolar renal lesion consistent with RCC (Figure 1). The peripheral blood smear and flow cytometry showed relative granulocytosis, 0.2% blasts, and no detected B cell/T cell malignancies or acute leukemias. A bone marrow biopsy confirmed no acute leukemia or lymphoma. Serum protein electrophoresis showed no M spike. Infectious serologies including *Mycoplasma pneumoniae*, *Ehrlichia*, Epstein-Barr virus, hepatitis panel, and human immunodeficiency virus were all negative.

He was started on methylprednisolone IV 1 mg/kg daily on hospital day one, IVIG 1 g/kg once daily for two days and folic acid 1 mg daily starting on hospital day three, followed by rituximab 375 mg/m2 once a week for four weeks starting on hospital day three. He was given a dose of epoetin alfa (EPO) 10,000 units on hospital day two.

**Figure 2:**
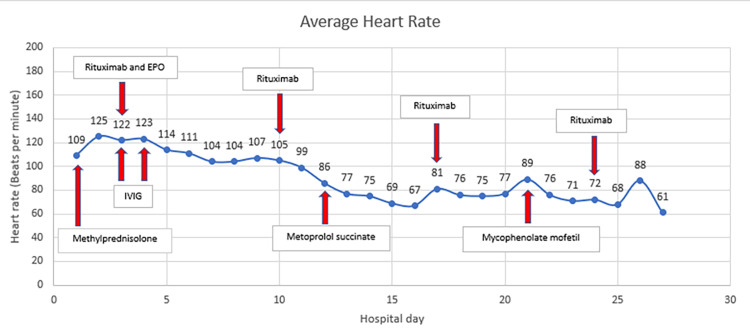
Average daily heart rate of the patient during entire hospitalization. Shown in the boxes with red arrows are treatments corresponding with the hospital day each was initiated. EPO (erythropoietin); IVIG (intravenous immunoglobulin)

**Figure 3:**
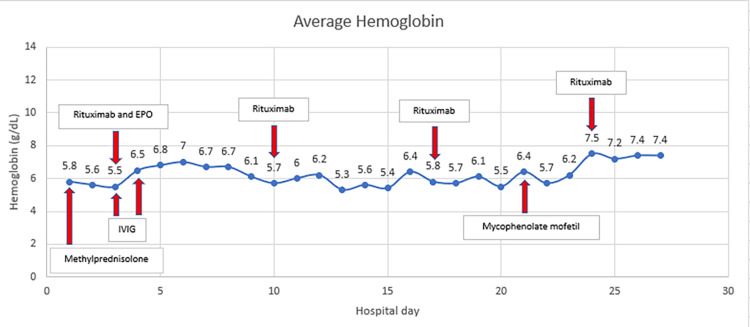
Average daily hemoglobin of the patient during entire hospitalization. Shown in the boxes with red arrows are treatments corresponding with the hospital day each was initiated. EPO (erythropoietin); IVIG (intravenous immunoglobulin)

Over the course of several days, he remained tachycardic and tachypneic, requiring blood transfusions at least every six hours to maintain hemoglobin around 6.0 g/dL. Figures 2, 3 show the trend of his heart rate and hemoglobin, respectively, with the immunosuppressive treatments shown on the hospital day each one was initiated. Despite aggressive transfusions and the early administration of high-dose IV methylprednisolone, his troponin increased to a peak of 8.75 ng/mL on hospital day three and his electrocardiogram (ECG) revealed sinus tachycardia rate 129 with a right bundle branch block that was new since his most recent ECG 10 months prior. An echocardiogram performed on hospital day two revealed an ejection fraction of 30%, severe global hypokinesis with regional variations, normal wall thickness, mild aortic stenosis, and peak pulmonary artery pressure was estimated to be 55 mmHg. The main competing etiologies for his heart failure were thought to be high output heart failure secondary to anemia versus acute coronary arterial thrombus.

Cardiology was consulted on hospital day three and determined that the patient was a poor candidate for cardiac catheterization due to his persistently high heart rates and ongoing requirements for blood transfusions. Goal-directed medical therapy was initiated with metoprolol succinate 25 mg daily on hospital day 12.

Hematology/oncology services were consulted on hospital day four when it became clear that our patient was not stabilizing with steroids or IVIG. Rituximab had already been started by the time this consult was made. The consultant recommended to maintain hemoglobin above 7 g/dL and hold transfusions if there were concerns for developing transfusion-associated circulatory overload (TACO) or transfusion-related acute lung injury (TRALI). Bone marrow biopsy was advised if there were to be no improvement in the next two weeks.

Over those two weeks, his hemolysis with symptomatic anemia persisted, although his average heart rate did improve to a rate less than 100 starting on hospital day 11. However, he was unable to reach a hemoglobin of 7 g/dL. Of note, the LDH level peaked at 1152 units/L on that day. The hematology/oncology consultant then recommended a lower transfusion threshold of 5-5.5 g/dL in order to decrease his risk of volume overload. He underwent a bone marrow biopsy on hospital day 15, the results of which are described above. Mycophenolate mofetil 500 mg twice daily was added on hospital day 21.

After 25 units of blood, methylprednisolone, EPO, IVIG, folic acid, rituximab, and mycophenolate mofetil, his hemoglobin finally improved to 7.5 g/dL on hospital day 24. The hematology/oncology consultant recommended monitoring him for the next 48 hours to ensure hemoglobin stability without further blood transfusions. After this criterion was met, he was discharged home with a hemoglobin of 7.4 g/dL on hospital day 27 with instructions to continue mycophenolate mofetil 500 mg twice daily, prednisone 120 mg daily, folic acid 1 mg daily, and metoprolol succinate 25 mg daily. Follow-up appointments with hematology/oncology and cardiology were made prior to discharge. An outpatient steroid taper was planned by the hematology consultant. 

## Discussion

WAIHA can present as a paraneoplastic syndrome in the setting of many different solid tumors. While extremely rare, one of the most common solid tumors associated with WAIHA is RCC [8]. In these cases, resection of the tumor is often curative of the hemolytic process. These patients are more likely to be unresponsive to steroid treatment prior to resection [8]. This lack of responsiveness was seen in our patient, who continued to require blood transfusions multiple times per day for roughly two weeks after initiating high-dose IV steroids.

The literature regarding WAIHA and RCC is quite limited. There are two case reports, one of which discusses WAIHA associated with renal urothelial cancer [9], and the other analyzes WAIHA associated with unspecified renal cell carcinoma [10]. Of these few cases of WAIHA secondary to RCC in the current literature, all patients experienced a complete remission of the WAIHA with resection of the tumor [9,10]. Since our patient was unable to undergo surgery, we were tasked with walking a balance between risking volume overload and transfusion reactions in the setting of newly reduced cardiac ejection fraction versus allowing his end-organ damage to worsen secondary to severe anemia. The addition of metoprolol succinate to his medication regimen likely augmented his ability to tolerate the lower hemoglobin level by artificially lowering his heart rate.

WAIHA can also develop with the use of certain drugs, most commonly antibiotics or chemotherapy agents. In approximately 15% of drug-induced WAIHA, non-steroidal anti-inflammatory drugs (NSAIDs) are thought to be the cause [11]. When drugs are the underlying cause, removal of the offending agent will result in hematological recovery within days [11]. Our patient admitted to daily ibuprofen use for headaches over the past month; however, his hemolysis did not improve with cessation of NSAIDs.

Typical treatment options for WAIHA proceed in a stepwise manner, starting with hemodynamic support in the form of blood transfusions and removal of the inciting cause, if possible. The retrospective study performed by Chen et al. determined that an ideal transfusion threshold in AIHA patients may be 4-5 g/dL [6]. According to this study, the lower threshold minimizes transfusion reactions and volume overload while attempting to achieve remission of the disease state. Some expert opinion suggests that the threshold should be between 6.0 to 10 g/dL, particularly in patients with severe symptoms [7]. Recommendations from the First International Consensus Group do not suggest a specific threshold, advising only that transfusions should not be delayed in severe or critical cases [12].

Our patient did not suffer any transfusion reactions during his hospitalization. However, he was at great risk for volume overload due to new onset heart failure with an ejection fraction of 30%. We struck a tenuous balance between ensuring timely replacement of his continuously depleting hemoglobin supply while closely monitoring his volume status with monitoring of vital signs and serial physical examinations. The addition of metoprolol succinate 25 mg daily on hospital day 12 likely provided a decrease in his myocardial oxygen demand and contributed to the successful avoidance of volume overload during his stay. Metoprolol was added at the recommendation of the cardiology service, who were consulted early in the hospital course. Prior to hospital day 12, he was determined to be unlikely to tolerate beta blockade due to concerns for the possibility of decreasing his blood pressure and worsening his critical state. 

After starting blood transfusions, the next step of treatment in WAIHA is administration of systemic glucocorticoids with 1-2 mg/kg dosing, with or without rituximab 375 mg/m2 IV once weekly for four weeks [5,12]. If there is little to no recovery within two weeks, antimetabolite immunosuppressants are added, which may include mycophenolate mofetil, cyclosporine, and azathioprine [12]. If there is minimal improvement after the implementation of these drugs, a splenectomy can also be considered. If splenectomy fails to alleviate hemolysis, alkylating agents such as cyclophosphamide or proteasome inhibitors such as bortezomib can be added [12]. Our patient received systemic glucocorticoids, rituximab, and mycophenolate mofetil, but splenectomy was not considered due to his persistent tachycardia and new onset heart failure.

In summary, we used increasingly potent immunosuppressive agents, hemoglobin production stimulating agents, and early consult to hematology/oncology and cardiology to determine the optimal threshold for blood transfusion to minimize complications. Overall, we found that using hemodynamic parameters to determine an appropriate transfusion threshold provided the most benefit. Despite reaching a stabilized hemoglobin level, our patient’s prognosis was very guarded upon discharge home because the underlying cause of his hemolytic anemia had still not been removed.

Given the lack of clinical trials investigating all types of AIHA, including WAIHA, most of the data used for treatment guidelines are gathered from case reports, case series, and expert opinion [12-13]. There is little to no guidance on treating WAIHA in cases where the underlying cause is identified but not removable. We hope that by relaying the events of this patient’s case we can shed more light on the more difficult aspects of treating WAIHA caused by solid tumors. 

## Conclusions

This was a case of treatment-resistant WAIHA likely secondary to RCC in a patient who was too hemodynamically unstable for tumor resection. He was treated with a total of 25 blood transfusions, IVIG, EPO, high-dose IV methylprednisolone, rituximab, and mycophenolate mofetil. The transfusion threshold for WAIHA is suggested to be as low as 4.0 g/dL and as high as 10 g/dL in the literature. However, there are very few recommendations regarding management of WAIHA secondary to unresectable solid tumors. Therefore, we based our transfusion threshold on heart rate and blood pressure parameters to decrease the likelihood of TACO, TRALI, and volume overload in the setting of heart failure with reduced ejection fraction. The addition of beta blockade with metoprolol decreased symptoms and stabilized heart rate.

In rare cases when the underlying cause of WAIHA cannot be treated, an individualized approach should be pursued. Prioritizing hemodynamic stability and mitigating the risk of end organ damage should be the primary goal. We recommend confirming a mean arterial pressure (MAP) of less than 65 or a heart rate greater than 100 to determine need for transfusion if the patient is not able to tolerate hemoglobin less than 5.0 g/dL.
